# Patient satisfaction and uptake of private-sector run malaria diagnosis clinics in a post-conflict district in Sri Lanka

**DOI:** 10.1186/1471-2458-14-641

**Published:** 2014-06-23

**Authors:** Deepika Fernando, Nipun Lakshitha de Silva, Isabella Ackers, Rabindra Abeyasinghe, Pandu Wijeyaratne, Senaka Rajapakse

**Affiliations:** 1 Department of Parasitology, Faculty of Medicine, University of Colombo, Kynsey Road, Colombo, Sri Lanka; 2 Tropical Medicine Research Unit, Faculty of Medicine, University of Colombo, Colombo, Sri Lanka; 3 Department of Public Health, University of Oxford, Oxford, UK; 4 World Health Organization, Port Moresby, Papua New Guinea; 5 Tropical and Environmental Diseases and Health Associates, Colombo, Sri Lanka

**Keywords:** Malaria, Surveillance, Patient, Satisfaction, Fever

## Abstract

**Background:**

With the incidence of malaria in Sri Lanka declining, intensive parasitological surveillance has been identified as a key strategy to achieve elimination by end 2014. Tropical and Environmental Diseases and Health Associates Private Limited (TEDHA) in collaboration with the Anti-Malaria Campaign established 43 malaria diagnostic laboratories (MDL) in four post-conflict districts of the Northern and Eastern Provinces. This study assesses the patterns of referral of patients with fever for malaria diagnosis by health care providers (HCPs) in four government hospitals in one of the districts of the Northern Province, and patient satisfaction with the laboratory services offered.

**Methods:**

In this prospective descriptive study, data was collected on the proportion of fever patients being referred by the HCP in hospitals for malaria screening, and the proportion thereof who underwent screening. An interviewer-administered questionnaire was also used to assess patient satisfaction among those attending MDL, which was graded on a scale of 0–4.

**Results:**

Of patients presenting to the hospitals with fever, only 44.3% were referred for malaria screening; 81.7% of them underwent screening. Referral depended largely on the presence of a permanent staff HCP. Satisfaction levels were high, with 86.55% giving an overall rating of 4. Comfort within the laboratory was rated satisfactory in three of the four hospitals.

**Conclusions:**

This study demonstrates the success of a public-private partnership in the malaria control programme in Sri Lanka. Malaria is considered low on the differential diagnosis in patients with fever even in previously malaria-endemic areas, due to the declining incidence of malaria and the increase in other febrile illnesses in these areas during the recent past. Private sector run malaria diagnostic services provided free of charge within government hospitals are viable and effective, and had good patient satisfaction ratings. In a country on the brink of eliminating malaria, there should be further emphasis on ensuring that HCPs refer patients for malaria diagnosis, in order to prevent a resurgence of the disease.

## Background

The annual parasite incidence of malaria in Sri Lanka has remained below 0.01 (total number of positive cases per 1000 risk population) since 2008 [1], with approximately 70% of malaria cases being reported from the Northern and Eastern provinces of Sri Lanka. In 2008, the National Anti-Malaria Campaign (AMC), with financial assistance from the Global Fund to fight AIDS, Tuberculosis and Malaria (GFATM) embarked on phased elimination of malaria from Sri Lanka through intensification of both parasitological and entomological surveillance [2]. The 27-year-old armed conflict in Sri Lanka resulted in a general breakdown of civil administration leading to hardships for communities in the Northern and Eastern Provinces [3,4]. Following the end of the conflict in 2009, with the reorientation of the national policy to malaria elimination mode, parasitological surveillance had to be intensified, with particular focus on these two provinces. In 2010, a total of 684 cases of malaria were reported from the whole of Sri Lanka, and 459 of these were from the Northern Province. In 2011, the numbers had diminished to 124 positive cases of malaria in the country, and 104 of these were from the Northern Province [1,3].

As a result of disruption of services in government hospitals in the region, the capacity of the AMC was inadequate to provide comprehensive malaria screening. This was in part due to inadequate diagnostic facilities, with laboratories available only in larger hospitals. In addition, there were shortages of trained staff, with many vacancies in cadre. Healthcare provision is nearly entirely by the public sector hospitals in these regions.

Tropical and Environmental Diseases and Health Associates Private Limited (TEDHA), a private sector organisation and one of the principal recipients of the GFATM grant, was requested to assist the AMC in strengthening malaria surveillance activities in the remotest areas in the districts of Ampara, Batticaloa, Trincomalee of the Eastern Province and the Mannar district of the Northern Province of Sri Lanka.

TEDHA carried out a two-pronged campaign of both active and passive case detection. For active case detection, mobile malaria clinics were conducted, where high-risk populations were screened for malaria parasites irrespective of whether they had symptoms or signs of malaria. The two key strategies employed by TEDHA for passive case detection were: a) the establishment of malaria diagnostic laboratories (MDL) in rural hospitals to provide malaria microscopy free of charge; and b) interventions to encourage healthcare providers (HCPs) to refer patients with fever for microscopic testing to confirm or rule out malaria. With the low malaria incidence reported and the disease on the brink of eradication, coupled with the rising incidence of other serious febrile illnesses such as dengue and leptospirosis, one of the problems identified during initial discussions was that malaria did not rank high in the differential diagnosis of fever, resulting in patients with fever not being routinely referred for malaria testing. To overcome this, TEDHA organised a series of face-to-face awareness programmes for the HCPs, encouraging them to refer all patients with fever to the MDL for free-of-charge malaria screening. TEDHA established MDLs in 43 rural hospitals in the four districts mentioned above, and trained personnel in malaria diagnosis. The MDL were manned by two individuals who were trained to prepare, stain, examine and report on thick and thin blood smears for malaria parasites. This was the first time in Sri Lanka that the private sector had established malaria diagnosis laboratories in government hospitals in Sri Lanka.

This study was carried out approximately one year after commencement of operations of the malaria elimination programme by TEDHA. The aims of this study were; a) to determine the proportion of patients presenting with fever being referred for malaria screening; b) to determine the proportion of such referred patients actually having malaria screening by blood smear; and c) to determine the satisfaction with the quality of services offered by the MDL.

## Methods

This was a cross-sectional study carried out over a period of four months beginning June 2011 in the post-conflict district of Mannar, located on the north-west coast of Sri Lanka in the Northern Province. This district has a square area of 2002 km^2^, and had a population of 95430 in 2011 [5]. The Mannar district was chosen for this study as it was an area most severely affected by the conflict, having been located on the border between government forces and rebels. Further, a large number of security personnel who are identified as a high-risk group for malaria were also based in this district. At the time the study commenced, road networks and reconstruction efforts were taking place in the district, and displaced persons were being relocated. TEDHA was responsible for malaria screening and diagnosis in four government hospitals (which were allocated to TEDHA by the AMC) which have been identified as A-D for the purpose of anonymity. TEDHA established fully equipped MDLs, and commenced screening for malaria in 2010. The study was conducted after a period of one year had elapsed after commencement of operations. Two persons were assigned to each laboratory, viz., A ‘Fever Surveillance Assistant’ who was responsible for taking a finger prick blood sample and a ‘Parasitological Surveillance Assistant’ who was responsible for staining and examining the blood smear.

We obtained the following data from hospital Out-Patients Department (OPD) records and records kept by HCPs and MDL: numbers of patients with history of fever attending the outpatients departments of the hospitals and the proportion thereof being referred to the MDL for malaria screening; the number of patients actually undergoing malaria screening at the MDL; and reasons for non-uptake (i.e., those not obtaining malaria screening despite being referred). In the OPD records, patient names with presenting complaints were recorded, with a number assigned. Records kept by HCPs had patients’ presenting complaint, and in cases of fever patients, whether referral for malaria diagnosis was done or not. The MDL maintains registers to obtain the name, age and sex of the patient presenting for blood smear examination. These registers are maintained in triplicate.

Further, we used an interviewer-administered pretested questionnaire to obtain information regarding satisfaction of patients presenting to the MDL, during the month of July. Satisfaction levels were determined from the time of arrival to the MDL to the point they left with the report. Satisfaction was determined under the following broad categories: a) sample collection (courtesy of the staff, procedure of taking consent, changing of gloves between patients, wearing of a laboratory coat); b) Comfort within the laboratory (over-crowding, seating arrangement, ventilation and temperature); and c) time taken to issue reports. A Likert scale of 0–4 was used, with 0 = “highly dissatisfied” to 4=“highly satisfied”, together with an open-ended section seeking views on methods of improving services. To minimize interviewer associated bias, all four interviewers involved in the study were trained on data collection over a period of one week and one of the Investigators visited a hospital each day to ensure data collection was taking place in line with the training given.

Sample size for this component of the study was estimated based on a population roughly equal to the number of patients presenting to the MDL during the preceding month of June. Based on a population size of 320, response distribution of 50%, confidence level of 95%, and margin of error of 5%, an estimated sample size of 170 was decided upon. Since randomisation was not possible, and given the limitations of time available to interview each patient, we chose to select alternate patients for the survey. On each day of the week the first patient was selected, and every alternate patient after that.

Socio-demographic data was obtained from all participants recruited for the study on satisfaction. The questionnaire was developed in English, translated to Tamil, and back-translated to English. Each of the hospitals was visited by one of the four interviewers on rotational basis on each day of the week, and the questionnaire was administered by a research assistant fluent in English and Tamil. Timings were made using a wall clock provided by TEDHA to assess the waiting time of interviewed patients. Data was analysed using STATA® statistical software. Descriptive data is presented as frequencies and percentages, and Chi-square test and Fisher’s exact test were used to identify determinants of satisfaction and uptake.

Patients who did not proceed for blood smear despite referral were identified at the hospital outpatient pharmacy. Pharmacists were advised to check from each patient for a prescription for blood smear given by doctor before dispensing drugs, and if there were any they were requested to meet the interviewer before leaving hospital. Reasons for non-uptake were also assessed by a pre-tested questionnaire with a list of possible reasons identified during preliminary interviews.

Informed verbal consent was obtained from all participants. Data were anonymised. Ethics approval was obtained from the Ethics Review Committee, Sri Lanka Medical Association.

## Results

### Number of patients with fever screened for malaria

Over a four month period, just 2140 (44.3%) of patients out of a total of 4834 patients presenting with fever were referred for blood smear examination for malaria diagnosis. Of these, 1748 (81.7%) visited the laboratory to be tested for malaria by microscopy, while the remainder left the hospital without undergoing the test (Table 1). All blood smears that were examined were negative for malaria parasites. There was, however, wide variation between the four hospitals in the percentages of patients with fever being referred for microscopic screening, ranging from 17.9% to 71.9% (Table 1). It was noteworthy that once referred, the likelihood of having a blood smear was similar in all four hospitals (78.8% to 85.5%) (Table 1).

**Table 1:** Number of OPD patients attending the hospitals and number who underwent blood smear examination between June-September 2011

**Hospital**	**Number with fever**	**Referred for microscopy**		**Patients in whom blood smear was done**	
		**Number**	**Percentage**	**Number**	**Percentage (of those referred)**
A	567	408	71.9%	329	80.6%
B	636	217	34.1%	171	78.8%
C	1896	1204	63.5%	981	81.5%
D	1735	311	17.9%	267	85.8%
Total	4834	2140	44.3%	1748	81.7%

The notation used by HCPs for referral for malaria diagnosis was variable; notations used included “MS” (malaria screening), “F” (fever), “PS” (parasitological screening) and “BT” (blood test). The use of different notations by HCP who were covering duty rather than the permanent staff HCP coincided with the lower success rates of referred patients reaching the malaria diagnosis laboratory, as hospital staff failed to recognise that certain notations indicated that malaria screening was required.

### Satisfaction of individuals who presented for blood smear examination with the TEDHA diagnostic facilities

During the month of July 2011, 393 individuals who presented to the four hospitals with fever were referred to the MDL for microscopy. Seventy eight percent (307/393) proceeded to the MDL to get their blood smears done. One hundred and nineteen individuals were interviewed after inviting 154, while the others did not give consent. Of the 86 who did not proceed to the MDL to undergo blood smear examination despite being referred by the HCP to do the test, 30 were interviewed.

The majority of those interviewed for satisfaction, i.e., 86.55% were highly satisfied (grading of 4 on the scale) with the overall services offered at the facility (Table 2). All of them indicated they would recommend the malaria diagnostic service to other members of their community. All (100%) indicated that the staff treated them with respect, were polite, and obtained consent prior to taking blood samples. Satisfaction on other aspects of the procedure of blood sampling was high (89.9% rating 4). Over 90% were satisfied with the comfort within the laboratory. However, the level of comfort, i.e., ventilation, seating arrangements etc., were significantly different between the hospitals. Hospitals A, C and D showed high satisfaction (93.2%) with regards to comfort of the laboratory, while just 37.5% at hospital B were satisfied with this aspect, and this was due to the small size of the room. The patients who were not satisfied with laboratory at hospital B indicated that the hospital should provide a better place to establish a malaria diagnosis laboratory as the room was too small, crowded and hot. Although over 50% of the patients (n = 64) reported a waiting time of over two and a half hours for their results, the majority, i.e., 89.9% stated that they were satisfied with the waiting time for results.

**Table 2:** Overall satisfaction levels scored for each of the TEDHA malaria diagnostic laboratories

**Hospital**	**Sample size**	**Highly satisfied n (%)**	**Somewhat satisfied n (%)**	**Neutral n (%)**	**Somewhat dissatisfied n (%)**	**Highly dissatisfied n (%)**
**A**	25 (21.01)	23 (92.00)	2 (8.00)	0 (0.00)	0 (0.00)	0 (0.00)
**B**	16 (13.45)	3 (18.75)	7 (43.75)	6 (37.50)	0 (0.00)	0 (0.00)
**C**	29 (24.37)	28 (96.55)	0 (0.00)	0 (0.00)	1 (3.45)	0 (0.00)
**D**	49 (41.18)	49 (100.00)	0 (0.00)	0 (0.00)	0 (0.00)	0 (0.00)

Three statistically significant determinants of overall patient satisfaction with the diagnostic service were found: awareness that the service is free; comfort of the laboratory; and waiting time. Prior awareness of the availability of the service was positively associated with higher satisfaction (p < 0.001) (Table 3).

**Table 3:** Association of service related factors and patients’ overall satisfaction with the TEDHA microscopy diagnostic service

**Determinants of overall satisfaction**	**Number (n = 119) n (%)**	**Outcome highly satisfied n (%)**	**Measure of association**	**p value**
**Hospital where screened**			χ^2^ = 3.693	<0.001
A	25 (21.01)	23 (92.00)		
B	16 (13.45)	3 (18.75)		
C	29 (24.37)	28 (96.55)		
D	49 (41.18)	49 (100.00)		
**Gender**			χ^2^ = 2.910	0.405
Male	51 (42.86)	42 (82.35)		
Female	68 (57.14)	61 (89.71)		
**Ethnicity**			Fishers Exact test	0.722
Sinhalese	1(0.84)	1 (100.00)		
Tamil	94 (78.99)	80 (85.11)		
Muslim	24 (20.17)	22 (91.67)		
**Highest Level of education completed**			Fishers Exact test	0.061
No formal education	6 (5.04)	4 (66.07)		
Primary	79 (66.39)	68 (86.08)		
Secondary or above	34 (28.37)	31 (91.18)		
**Aware that the service is free**			χ^2^ = 22.721	<0.001
Yes	112 (94.12)	102 (91.07)		
No	7 (5.88)	1 (14.29)		
**Satisfaction level with comfort of the laboratory**			χ^2^ = 8.291	<0.001
Highly satisfied	99 (83.19)	95 (95.96)		
Somewhat satisfied	10 (8.40)	6 (60.00)		
Neutral	7 (5.88)	1 (14.29)		
Somewhat dissatisfied	3 (2.52)	1 (33.33)		
Highly dissatisfied	0 (0.00)	0 (0.00)		
**Satisfaction with waiting time**			χ^2^ = 3.819	<0.001
Highly satisfied	90 (75.63)	87 (96.67)		
Somewhat satisfied	17 (14.29)	15 (88.24)		
Neutral	9 (7.56)	1 (11.11)		
Somewhat dissatisfied	2 (1.68)	0 (0.00)		
Highly dissatisfied	1 (0.84)	0 (0.00)		

Socio-demographics did not play a significant role in influencing overall patient satisfaction, with no difference in the level of satisfaction between genders (P = 0.405), ethnic groups (P = 0.722), or age (P = 0.590). Although the relationship to level of education just failed to reach significance (P = 0.061), lower levels of education showed a trend towards being associated with lower levels of satisfaction with the overall service.

The majority of the patients (64.7%) complained about the restricted opening times of the hospitals limiting the ability for them to collect the result. This was particularly noted for weekends as it was common for patients tested on a Saturday to collect their reports on the following Monday. A system to enable patients to get results outside opening hours was suggested.

Among those who did not visit the laboratory despite being referred for testing, the common reasons given for not undergoing the test were: lack of transport to get to their houses if it got late (46.7%); malaria not being common in their area and thereby them not seeing a necessity to have a their blood taken (40%); and the laboratory being too crowded (13.3%).

## Discussion

The GFATM has recognized the importance of partnership between civil society, private sector and government agencies to ensure the success of disease control efforts, as a means towards faster implementation and long term sustainability [6]. In order to pool knowledge and resources, and to combine the different strengths of the public and private sector, the AMC of the Ministry of Health established a partnership with TEDHA Private Limited to strengthen malaria surveillance in the country. Since January 2010, TEDHA has supported the AMC with technical expertise and intensified surveillance activities in designated areas in the Eastern Province and Mannar district of the Northern Province of Sri Lanka. The high levels of satisfaction as well as high uptake of MDL established by TEDHA in government hospitals demonstrates the success of a public-private partnership towards malaria elimination. However, the relatively low rates of referral for malaria screening by HCPs is a cause for real concern, and is likely to hinder thrust strategies aimed at eliminating the disease. The Northern Province, including the Mannar District, reported the highest number of malaria infections, with 124/808 cases being reported from the Mannar district alone during 2010/2011 [3]; thus inability to screen fever patients for malaria parasites is likely to hinder Sri Lanka’s efforts in the malaria elimination strategy. Several causes for this low rate of referral can be postulated. Firstly, the rise in prevalence of other febrile illnesses, such as dengue, leptospirosis and typhus, and in particular widespread publicity given towards identification of dengue could have resulted in diminished interest in malaria as a cause of fever [7]. Secondly, a decline in malaria cases could potentially have resulted in diminished focus on malaria related practices, especially among newly graduated HCPs [8]. Such a trend worryingly echoes the mistakes of 1963 in Sri Lanka, where a decline in vigilance and sustained parasitological surveillance resulted in a massive resurgence of the disease in the following decade [9]. With the country on the brink of malaria elimination, a repetition of this mistake should be avoided at all costs. Ensuring that 100% of fever patients in these previously malaria endemic districts are referred for screening is of critical importance. This message has since been communicated very clearly to HCPs in these areas.

Lack of standardized criteria for referral for malaria screening has been suggested to be an important factor leading to low testing rates [10]. In countries with high prevalence of malaria, health care personnel have a tendency to treat cases of fever with antimalarials, without testing for malaria [11,12], even though this is contrary to guidelines [13]. Healthcare personnel in these countries have a low threshold for diagnosis and treatment of malaria, and hence they often treat without investigations, resulting in low testing rates. The opposite of this phenomenon occurs in formerly endemic countries in which malaria is on the brink of elimination. Training and awareness programmes have shifted the focus away from malaria towards other infectious diseases; thus testing rates are low simply because the threshold for diagnosis of malaria is high. This highlights the fact that problems associated with low uptake are unique to different settings.

The use of non-standard abbreviations and notations by HCPs when referring patients to MDL resulted in lower uptake. To rectify this, TEDHA distributed a prescription pad with instructions to use the universal notation MP (malaria parasite) to all HCPs in the region. An awareness programme was also conducted amongst the hospital staff regarding the need to direct patients towards the TEDHA MDL. Pharmacists were also advised to check whether blood smear examination has been carried out prior to issuing drugs. It is postulated that if these weaknesses can be rectified, screening of malaria could reach 100%.

Patient satisfaction is an important measurement of health care, and also an important determinant of uptake of screening services; this is a unique instance of a screening service carried out by a private sector organisation within the government hospitals. Socio-demographics had no role in influencing the overall level of satisfaction with the process of malaria screening carried out at the hospitals. This suggests that the recommendations for improvement are universal and should be applied throughout TEDHAs diagnostic services.

The comfort of the laboratory plays an important role in determining a patients’ level of satisfaction with the microscopic diagnosis service. One of the laboratories in particular was considered particularly uncomfortable, as the room was very small and hot. Following the completion of this study, this laboratory has been relocated to an alternative room in the hospital with more room and proper ventilation to ensure patient comfort. However, the high level of overall satisfaction with regard to the entire blood taking process and issuing of reports on time indicates that a proper service is being provided by the MDLs.

The lack of awareness amongst the community that microscopy services are available free of charge has led to TEDHA carrying out awareness programmes with the assistance of its community mobilisation officers (CMOs). Emphasis is being placed on the availability of malaria diagnostic services at the four government hospitals in the Mannar district, through lectures delivered at schools and to persons with high risk of contracting malaria, such as security personal and by distribution of leaflets and posters.

The short opening times on Saturday corresponds to the working hours of the government doctors (8.30-1.00 pm). This results in some patients having difficulty collecting their diagnosis cards, particularly when they have to travel from far. A solution is being explored to enhance collaboration with the hospitals with wards which are open throughout the weekend so as to enable the patients to collect the reports from the outpatients department. This would prevent patients being tested on Friday evening or Saturday morning having to wait till the following Monday to receive their diagnosis.

From the data collected it is also evident that many fever patients are being lost during the passive screening referral process. This was due to many reasons such as lack of transport back to their homes if a delay occurred, not having sufficient time to get a blood smear examined or not understanding the importance of malaria diagnosis. The wait of an average time of 2.5 hours to get the results of blood smear examination could have contributed to individuals not getting their blood smear examination. However, following this study, changes were made to the protocols in blood smear preparation, reducing the time required to issue a report to less than 1 hour in over 80% of the cases [14]. Most of the patients had to depend on public transport to reach the health care facilities as they were not within walking distance. At the time the study was being carried out, public transport facilities were being improved in this post-conflict district and was not readily available.

Our study has certain other limitations. The study interviewed only those participants who gave consent to be interviewed and were therefore more likely to be satisfied with the service on offer. Furthermore, the relatively small sample size (N = 119) limited the extent of statistical analysis that could be performed. However, the study on satisfaction amongst the participants was carried out by an independent interviewers and not employees of TEDHA. The confounding effect resulting from the presence of the study investigators within the laboratory on the laboratory staff, potentially influencing their behaviour and practice is another limitation. However, space and human resources were limited, and interviewing the patients in the laboratory premises was the only pragmatic method. For logistic reasons we were able to conduct this study in just one of the four districts where TEDHA’s intervention strategies were in place. While acknowledging that there could be variation in the results of these interventions in the other districts due to a variety of factors, our study did focus on the region most severely affected by the war, thus showing the effectiveness of such strategies in a post-conflict zone. Another limitation of this study is that we did not obtain details of gender or ethnicity of all patients presenting to OPDs with fever, as it was not logistically feasible.

Overall, this study highlights how the private sector can contribute in a number of ways to supplement national health programmes as this is the first time in Sri Lanka that the private sector has successfully established malaria diagnostic facilities free of charge in government hospitals for malaria diagnosis. It is well known that, in some situations, the private sector has a better capacity and ability to reach populations and provide services which cannot be accomplished by government or civil society. Areas in which the private sector can contribute include the provision of technical expertise, human resources development, strengthening health information systems and ensuring that other critical gaps are addressed. This role becomes more important in countries in which the private sector is a major player in the delivery of health care.

## Conclusions

Malaria is considered low on the differential diagnosis in patients with fever by HCPs, resulting in low levels of referral for malaria screening, even in previously high malaria endemic areas in Sri Lanka. We postulate that this is due to the sharp decline in the incidence of malaria seen in these areas during the recent past. However, a low threshold for referral for malaria screening is essential in the push towards malaria elimination, and also to prevent a resurgence of the disease. Creating awareness among healthcare personnel on importance of close surveillance as well as building up liaison between medical and laboratory staff is of utmost value in achieving close parasitological surveillance. Private sector run malaria diagnostic services within government hospitals are viable and effective, especially where government sector is unable to accomplish required standards on its own. Good patient satisfaction ratings on their services help to maintain better uptake of the facilities by the patients. In a country on the brink of eliminating malaria, intensive surveillance is essential to prevent a resurgence of the disease.

## Abbreviations

AMC: Anti malaria campaign; CMO: Community Mobilization Officer; GFATM: Global fund for elimination of AIDS, Tuberculosis and Malaria; HCP: Health care personnel; MDL: Malaria diagnostic laboratory; OPD: Out Patient Department; TEDHA: Tropical and Environmental Diseases and Health Associates.

## Competing interests

All authors declare that they have no competing interests.

## Authors’ contributions

DF, PW and RA designed the study, wrote the proposal and got ethical approval and funding to carry out the study. IA was the research student who collected the data. NLDS assisted in data entry and data analysis. DF and SR interpreted the results and initiated writing of the manuscript. All authors contributed towards writing of the manuscript and approved the final version.

## Pre-publication history

The pre-publication history for this paper can be accessed here:

http://www.biomedcentral.com/1471-2458/14/641/prepub
